# Improvement of fibroblast growth factor 21 resistance probably confers the beneficial effects of acute exercise

**DOI:** 10.14814/phy2.12946

**Published:** 2016-09-15

**Authors:** Li‐Ying Miao, Ri‐Yue Jiang, Bin Zhu

**Affiliations:** ^1^ The Blood Purification Center The Third Affiliated Hospital of Soochow University Changzhou China; ^2^ Department of Radiation Oncology The Third Affiliated Hospital of Soochow University Changzhou China; ^3^ Department of Critical Care Medicine The Third Affiliated Hospital of Soochow University Changzhou China

## Abstract

FGF21 resistance improvement, but not solely alteration of FGF21 expression, would probably facilitate the beneficial effects of acute exercise.

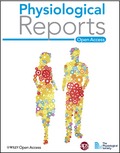

Dear Editor

It has been widely known that physical exercise may benefit a lot to the physiological functions. Recently, we read with great interest the paper by Tanimura et al. (2016) entitled “Acute exercise increases fibroblast growth factor 21 in metabolic organs and circulation”, in which the authors who conducted an excellent study including human subjects and animals concluded that a significantly up‐regulation of fibroblast growth factor (FGF) 21 is physiologically triggered by acute exercise in serum. We do not totally agree with this conclusion, and would like to put forward an opinion about the improvement of FGF21 resistance in the acute exercise.

FGF21 is a protein predominately secreted by liver, adipose tissues and pancreas that could stimulates glucose uptake (Otero et al. 2014). Currently, increasing evidence suggests that FGF21 as a novel adipokine has physical potential to increase energy expenditure (Itoh 2014). In this study, the authors of Tanimura et al. (2016) got a conclusion that increased FGF21 levels may underlie the favorable effects of acute exercise. However, we suggest that excessively up‐regulated FGF21 may induce a vicious loop of FGF21 resistance. FGF21 resistance could be likely described as an imbalance between FGF21 and its occupied receptors. That is, FGF21 receptors such as FGFR1c and/or FGFR4 could not supply sufficient binding sites for FGF21 (Fisher et al. 2010). In this regard, FGF21 resistance improvement, but not solely alteration of FGF21 expression, would probably facilitate the beneficial effects of acute exercise. Further studies on the physiological role of FGF21 in the acute exercise are required.

## Conflicts of Interest

The authors declared no any potential conflicts of interest.

## References

[phy212946-bib-0001] Fisher, F. M. , P. C. Chui , P. J. Antonellis , H. A. Bina , A. Kharitonenkov , J. S. Flier , et al. 2010 Obesity is a fibroblast growth factor 21 (FGF21)‐resistant state. Diabetes 59:2781–2789. 20682689 10.2337/db10-0193 PMC2963536

[phy212946-bib-0002] Itoh, N . 2014 **FGF21** as a Hepatokine, Adipokine, and Myokine in Metabolism and Diseases. Front. Endocrinol. (Lausanne) 5:107. 25071723 10.3389/fendo.2014.00107 PMC4083219

[phy212946-bib-0003] Otero, Y. F. , T. M. Lundblad , E. A. Ford , L. M. House , and O. P. McGuinness . 2014 Liver but not adipose tissue is responsive to the pattern of enteral feeding. Physiol. Rep. 2:e00250. 24744913 10.1002/phy2.250 PMC3966249

[phy212946-bib-0004] Tanimura, Y. , W. Aoi , Y. Takanami , Y. Kawai , K. Mizushima , Y. Naito , et al. 2016 Acute exercise increases fibroblast growth factor 21 in metabolic organs and circulation. Physiol. Rep. 4:e12828. 27335433 10.14814/phy2.12828 PMC4923231

